# Pigment Nephropathy: Novel Insights into Inflammasome-Mediated Pathogenesis

**DOI:** 10.3390/ijms20081997

**Published:** 2019-04-23

**Authors:** Kurt T. K. Giuliani, Andrew J. Kassianos, Helen Healy, Pedro H. F. Gois

**Affiliations:** 1 Kidney Health Service, Royal Brisbane and Women’s Hospital, Brisbane, QLD 4029, Australia; Kurt.Giuliani@uqconnect.edu.au (K.T.K.G.); Andrew.Kassianos@qimrberghofer.edu.au (A.J.K.); Helen.Healy@health.qld.gov.au (H.H.); 2 Conjoint Kidney Research Laboratory, Chemical Pathology—Pathology Queensland, Brisbane, QLD 4029, Australia; 3 Faculty of Medicine, University of Queensland, Brisbane, QLD 4006, Australia; 4 Institute of Health and Biomedical Innovation/School of Biomedical Sciences, Queensland University of Technology, Brisbane, QLD 4059, Australia

**Keywords:** rhabdomyolysis, pigment nephropathy, haem, NLRP3 inflammasome, acute kidney injury

## Abstract

Pigment nephropathy is an acute decline in renal function following the deposition of endogenous haem-containing proteins in the kidneys. Haem pigments such as myoglobin and haemoglobin are filtered by glomeruli and absorbed by the proximal tubules. They cause renal vasoconstriction, tubular obstruction, increased oxidative stress and inflammation. Haem is associated with inflammation in sterile and infectious conditions, contributing to the pathogenesis of many disorders such as rhabdomyolysis and haemolytic diseases. In fact, haem appears to be a signalling molecule that is able to activate the inflammasome pathway. Recent studies highlight a pathogenic function for haem in triggering inflammatory responses through the activation of the nucleotide-binding domain-like receptor protein 3 (NLRP3) inflammasome. Among the inflammasome multiprotein complexes, the NLRP3 inflammasome has been the most widely characterized as a trigger of inflammatory caspases and the maturation of interleukin-18 and -1β. In the present review, we discuss the latest evidence on the importance of inflammasome-mediated inflammation in pigment nephropathy. Finally, we highlight the potential role of inflammasome inhibitors in the prophylaxis and treatment of pigment nephropathy.

## 1. Introduction

Haem complexes consist of an Fe atom which is coordinated within the centre of a heterocyclic ring known as a protoporphyrin [1]. Haem-containing proteins are a large class of metalloproteins that play a pivotal role in maintaining basic biological functions [2]. Their broad activities range from mitochondrial electron transfer, oxygen transport and storage to signal transduction and control of gene expression [2]. 

Among the different haem group variants, haem a, b and c are the main biological types [3,4]. Of the haem variants, haem b is the most abundant form and is present biologically within myoglobin and haemoglobin, whilst haem a and c are present in cytochromes. Haem function as a prosthetic group in haemoproteins and are essential for reversible oxygen binding and transport [5,6]. However, under pathological conditions, an excess of circulating free haem may be highly cytotoxic and result in tissue damage, including within the kidney [3,6]. 

Pigment nephropathy (PN) is an acute decline in kidney function following the breakdown and deposition of endogenous haem pigment-containing proteins (myoglobin, haemoglobin) within renal tissue [7]. Both myoglobin and haemoglobin are freely filtered by glomeruli and when oxidised, release their haem moiety into the urinary space [8,9]. However, within the nephron, excess haem pigments may cause renal vasoconstriction, tubular obstruction, increased oxidative stress and inflammation [10,11,12,13]. 

Inflammation is an essential response of the innate immune system to harmful stimuli [14]. Haem is associated with inflammation in sterile and infectious conditions, contributing to the pathogenesis of many disorders such as rhabdomyolysis and haemolytic diseases [15]. There is an increasing body of evidence that haem trigger the inflammasome signalling cascade and ultimately, the innate immune response [16,17].

In the present review, we discuss the potential role of inflammasome activation as a driver of inflammation in PN. We explore the rationale of translating small molecule inhibitors of inflammasome activation already in clinical use, for diseases outside the kidney, in the prevention and treatment of PN.

## 2. The Nucleotide-Binding domain-Like Receptor Protein 3 (NLRP3) Inflammasome

The inflammasomes are a family of cytosolic signalling complexes with a central role in the activation of innate immune responses via the maturation and secretion of pro-inflammatory cytokines (interleukin (IL)-1β and IL-18) [18]. In particular, the nucleotide-binding domain-like receptor protein 3 (NLRP3) inflammasome, an extensively characterized inflammasome family member, is widely implicated in a variety of renal injuries, including acute and chronic kidney disease (CKD) [19,20,21]; oxalate and uric acid crystal nephropathy [22,23]; and diabetic nephropathies [24]. Inflammasomes respond to a diverse range of pathogen-associated molecular patterns (PAMPs) and endogenously derived damage-associated molecular patterns (DAMPs) via a suite of pattern recognition receptors (PRR). Of particular note, endogenous particulate matter, such as haem [16,17], monosodium urate (MSU) [25], oxalate [23,26] and cholesterol crystals [27,28] have all been identified as potent triggers of NLRP3 inflammasome activation and the subsequent release of pro-inflammatory cytokines [24]. 

Recently, Liston and Masters [29] proposed a mechanism of inflammasome activation in addition to the PAMP-DAMP axis. This mechanism responds to a loss of homeostasis via ‘homeostasis-altering molecular processes’ (HAMPs). They hypothesized that the PAMP-DAMP-HAMP axis was, collectively, likely to be sufficient for effective immunity and that deficiencies in this axis may cause the pathological inflammatory activation observed in sterile injury [29]. Examples of HAMPs which activate the inflammasome are perturbed membrane potential through K^+^ efflux and Ca^2+^ influx [30], extracellular adenosine triphosphate (ATP) [31,32,33], and mitochondrial damage through reactive oxygen species (ROS) [34], altered mitochondrial membrane potential (ΔΨ_m_) [35] and oxidised mitochondrial DNA (mtDNA) [36]. While their activation triggers may be diverse, the signalling pathways of inflammasome activation can be categorized into either canonical or non-canonical activation.

### 2.1. Canonical Inflammasome Activation

**Figure 1:**
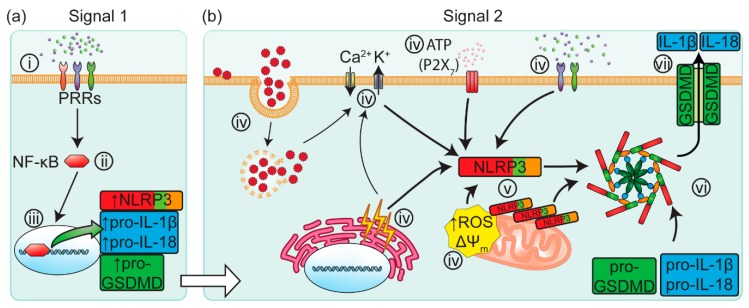
The canonical inflammasome activation signalling cascade is initiated by signal 1 PAMPs and DAMPs. (**a**) Signal 1 elicits the activation of PRRs on the cell surface (i). The activation of PRRs results in a downstream signalling cascade, triggering the translocation of NF-κB into the nucleus (ii), where NF-κB upregulates the expression of NLRP3, pro-GSDMD, pro-IL-1β and pro-IL-18 (iii). (**b**) Signal 2 is provided by an array of PAMPs, DAMPs and HAMPs (iv), including arrested phagocytosis, perturbed membrane potential (ΔΨ_m_), endoplasmic reticulum stress, extracellular ATP, and mitochondrial dysfunction. NLPR3 proteins which have co-localized to the mitochondria (v) are ideally located to rapidly respond to these markers of cellular stress. NLRP3 then oligomerizes with ASC and pro-Caspase-1, forming the NLRP3 inflammasome complex (vi). Caspase-1 undergoes self-cleavage whilst bound to the inflammasome complex (vi), driving the post-translational processing of IL-1β, IL-18 and GSDMD. Once cleaved, GSDMD proteins self-oligomerize to form pores in the cell membrane (vii), allowing for the rapid release of IL-1β and IL-18. In addition, these GSDMD pores may also drive cell-death via pyroptosis. ASC: Apoptosis-associated Speck-like protein containing a Caspase-activation-and-recruitment domain; PRR: pattern recognition receptor; PAMP: pathogen-associated molecular pattern; DAMP: damage-associated molecular pattern; nuclear factor kappa-light-chain-enhancer of activated B cells: NF-κB; NLRP3: nucleotide-binding domain-like receptor protein 3; IL: interleukin; GSDMD: Gasdermin D; ROS: reactive oxygen species; ΔΨ_m_: mitochondrial membrane potential.

Following the detection of PAMPs or DAMPs (Signal 1) by PRRs, the NLRP3 inflammasome is canonically activated in an orchestrated cascade of signals [37], see Figure 1. The transmembrane protein family of Toll-like receptors (TLRs) play an important role as PRRs, activating the downstream signalling cascade. This signalling cascade is known as the “priming” phase of inflammasome activation. Once primed, the nuclear factor kappa-light-chain-enhancer of activated B cells (NF-κB) signalling complex translocates to the cell nucleus where it promotes the upregulation of NLRP3 and immature forms of IL-1β and IL-18 [38]. 

Following the priming phase, a second signal (Signal 2) is required to elicit the activation of the inflammasome, see Figure 1b. These signals can include interrupted phagocytosis [39], extracellular ATP [31,32,33], K^+^ and Ca^2+^ flux [39,40,41], endoplasmic reticulum stress [42], mitochondrial ROS [34], ΔΨ_m_ [35] and the release of oxidised mtDNA [36]. Particulate matter are also potent secondary signals which can activate the NLRP3 inflammasome via cell-surface contact [39]. The mechanism for detection of these PAMP/DAMP/HAMPs by NLRP3 remains poorly understood.

Once activated by these molecular signalling patterns, NLRP3 proteins self-oligomerize and nucleate the formation of the NLRP3 inflammasome complex. This inflammasome complex consists of the NLRP3 protein, the ASC (Apoptosis-associated Speck-like protein containing a Caspase-activation-and-recruitment domain) adaptor protein and pro-caspase-1. Boucher, et al. [43] recently showed that pro-caspase-1 proteins dimerize following their recruitment to the inflammasome complex, before self-cleaving into an active state. The transiently active caspase-1 dimer undergoes additional cleavage, forming a proteolytically active holoenzyme with the inflammasome, capable of processing the pro-inflammatory cytokines IL-1β and IL-18 into their active forms [18,43]. Caspase-1 also cleaves Gasdermin-D (GSDMD) into its active form. Active GSDMD translocates to the cell membrane and forms GSDMD pores in the plasma membrane, driving pyroptosis and the consequent rapid release of IL-1β and IL-18 into the surrounding extracellular micro-environment [44,45,46,47,48]. 

### 2.2. Non-Canonical Inflammasome Activation

Non-canonical activation of the inflammasome differs in that it is dependent on caspase-11 (murine) or caspase-4 (human) activity [49,50,51]. Gram-negative bacteria-derived PAMPs are established triggers of non-canonical activation, directly sensed by and activating caspase-11/-4 [51]. Active caspase-11/-4 proteolytically cleave pro-GSDMD into its active state, effecting cell death by pyroptosis [49,50]. Kayagaki, et al. [50] showed that murine caspase-11 also triggers an NLRP3-inflammasome response through an as-yet-to-be identified mechanism, resulting in the release of IL-1β and IL-18 [50]. In humans, caspase-4 is required for the maturation and release of IL-18 via a non-canonical inflammasome pathway [51]. However, the role of non-canonical inflammasome activation in kidney disease remains to be elucidated. 

### 2.3. Inflammasomes in the Kidney

Inflammasome activation is a key driver of the pathobiology in a variety of murine models and human etiologies of acute kidney injury (AKI) and CKD. Several murine studies investigating NLRP3 function, using small-molecule inflammasome-specific inhibitors or gene knockout models, have provided strong evidence for inflammasome activity in renal tissue injury. Specifically, *Nlrp3^-/-^, Asc^-/-^* and *Casp1^-/-^* knock-out models have less kidney tissue damage and disease phenotype in unilateral ureteral obstruction (UUO) [52,53], diabetic kidney disease (DKD) [54] and crystal nephropathy [23,26]. However, the PAMPs/DAMPs/HAMPs that trigger inflammasome activation in these models are under active investigation.

Elevated soluble uric acid levels have been reported in the obstructed kidney of UUO mice [53]. Uric acid is an established activator of the inflammasome [55]. Furthermore, ROS derived from the activity of xanthine oxidase (XO), an enzyme which produces uric acid via purine catabolism, has also been reported to elicit an inflammasome response [56]. Allopurinol is a widely prescribed pharmaceutical used in the treatment of gout and directly inhibits XO activity. Notably, UUO mice treated with allopurinol exhibit less NLRP3 and IL-1β expression within the UUO kidney compared to untreated UUO controls [53]. These studies suggest a dual protective role for allopurinol by inhibiting both uric acid production and XO activity, thus preventing inflammasome activation. 

Shahzad, et al. [54] reported NLRP3 activation in podocytes, an important cell type in the glomerular filtration barrier, in a murine DKD model [54]. Interestingly, this study demonstrated increased IL-1β and IL-18 expression within plasma and renal cortical extracts of diabetic animals, correlating with the functional kidney biomarker urine albumin/creatinine ratio [54].

IL-1β and IL-18 are produced by infiltrating hematopoietic cells, such as dendritic cells (DC) and macrophages, in mouse kidneys [57]. Supporting this concept, DC depletion in a crystal-induced model of murine renal fibrosis, resulted in reduced fibrosis and improved kidney function [20]. Furthermore, a similar outcome was achieved by treatment with a specific small molecule NLRP3 inflammasome inhibitor (MCC950; detailed below in Section 6.1) that blocked NLRP3 activation in kidney DC, reduced IL-1β and IL-18 production and inhibited the progression of renal fibrosis [20].

In contrast to these murine studies, the examination of inflammasome-mediated renal pathology in humans is less extensive. Whilst human proximal tubular epithelial cells (PTEC) appear to have the necessary inflammasome-related machinery, there is a paucity of evidence for its activation, particularly, whether these cells secrete IL-1β and IL-18 [58]. Intriguingly Kim, et al. [58] recently described an inflammasome-independent role for NLRP3 in human PTEC. In this study, hypoxic injury to PTEC increased NLRP3 expression independent of ASC, caspase-1, and IL-1β. Instead, the NLRP3 protein bound to the mitochondrial antiviral signal (MAVS), resulting in mitochondrial dysfunction (increased mitochondrial ROS) and cell death [58]. There is also emerging evidence that human tubular cells in acute oxalate nephropathy undergo a form of regulated cell death termed necroptosis. Products of necroptosis include DAMPs with the capacity to activate the canonical inflammasome pathway in innate immune cells (DC, macrophages) within the tubulointerstitium [20]. Our group has indeed shown increased numbers of activated human DC within the tubulointerstitium of fibrotic kidney biopsies, accumulating adjacent to injured PTEC [59]. 

The kidneys play a major role in maintaining homeostasis and regulating blood pressure. Renal inflammation and fibrosis are well-known contributing factors in the pathogenesis of hypertension [60]. In a murine model of salt-induced hypertension, NLRP3 inhibition by treatment with MCC950 reduced hypertension and heart rate, in addition to reduced inflammasome priming, inflammatory cytokines, kidney immune cell infiltration and kidney fibrosis [60]. Nevertheless, the specific mechanisms by which the inflammasome contributes to systemic hypertension are still unclear. Furthermore, the inflammasome-dependent interactions between specialized renal parenchymal and innate immune cells, in particular, the role of NLRP3 signalling in driving the pathobiology of human PN, remains to be elucidated.

## 3. Haem Catabolism and Role in Immune-Mediated Pathology

Excess haem pigments are highly cytotoxic in the kidney, leading to oxidative stress and inflammation under injurious conditions [61,62]. Our understanding of immune-mediated pathological conditions is that oxidative stress and inflammation are interdependent processes rather than discrete pathways of injury [63].

**Figure 2:**
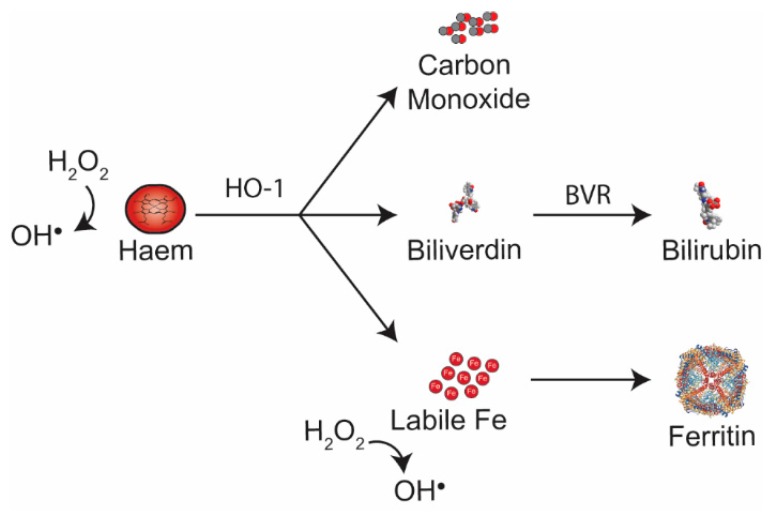
Haem catabolism by HO-1 produces equimolar amounts of carbon monoxide, Biliverdin and labile iron. Biliverdin is converted to bilirubin by biliverdin reductase. Labile Fe can produce ROS, but is rapidly bound to ferritin. Ferritin (PBD ID: 5Z8U) image generated using the RCSB PDB NGL viewer [67]. BVR: Bilirubin reductase; HO-1: Haem oxygenase-1.

Free haem catalyses the formation of highly toxic free radicals—hydroxyl radicals (OH∙)—from hydrogen peroxide (H_2_O_2_) via the Fenton reaction. Under homeostatic conditions, excess free cellular haem is catabolized by haem oxygenases (HO)—stress-responsive HO-1 and constitutive HO-2, as summarized in Figure 2. Catabolism of free haem by HO leads to the production of: (1) carbon monoxide (CO); (2) biliverdin (BV), that is converted by biliverdin reductase (BVR) to the antioxidant bilirubin; and (3) the release of labile Fe, which is promptly bound to ferritin (FtH), collectively preventing cellular oxidative stress [64,65,66]. However, under pathological conditions, the accumulation of intracellular free haem can exceed the rate of haem degradation by the HO-1 isoenzyme. Furthermore, levels of cellular Fe can be greater than the scavenging capacity of FtH. When this occurs, free haem and/or labile Fe accumulate in cells and drive oxidative stress in the micro-environment. The uncontrolled generation of free radicals and the subsequent imbalance between reactive metabolites and endogenous anti-oxidants constitutes the stress response and ultimately lead to cellular damage and inflammation.

Haem directly regulates inflammatory leukocyte migration and retention in vitro and in vivo [68]. In rodent models, intraperitoneal and intrapleural injection of haem results in dose-dependent neutrophil migration into the respective body compartments [68,69]. Haem inhibits neutrophil apoptosis, resulting in the accumulation of neutrophils at sites of haem deposition, and drives expression of proinflammatory cytokines [69,70,71]. Haem has also been reported to induce surface expression of adhesion molecules—i.e., intercellular adhesion molecule-1 (ICAM-l), vascular adhesion molecule-1 (VCAM-l) and endothelial leukocyte adhesion molecule (E-selectin)—in human endothelial cells, thereby driving the adhesion/retention of leukocytes [72]. 

Recent evidence suggests haem can trigger activation of innate immune cells via the NLRP3 inflammasome. Dutra et al. showed that haem activation of the NLRP3 inflammasome in bone marrow macrophages was dependent on NADPH oxidases, K^+^ efflux and generation of mitochondrial ROS [8]. Notably, NLRP3 activation was independent of haem internalization, lysosomal damage and cell death [8]. Inflammasome activity within immortalized human endothelial cells in response to haem has also been reported in vitro, where haem was sufficient to induce significantly increased IL-1β mRNA transcripts and cytokine release [16]. Intriguingly, HO-1 activity appears to attenuate NLRP3 activity. However, this may be an indirect consequence of haem catabolism by HO-1, rather than direct interactions between HO-1 and NLRP3 [73]. Although recent studies suggest haem is an important trigger of the canonical inflammasome pathway [8,73,74], its functioning via non-canonical NLRP3 inflammasome activation in renal cells has not been explored.

## 4. Myoglobin-Mediated Pigment Nephropathy

Rhabdomyolysis is a clinical syndrome following physical, thermal, toxic, metabolic, ischaemic, infective and inflammatory insults to muscles [13]. The final step of the skeletal muscle breakdown is the release of toxic intracellular components, such as the hemoprotein myoglobin, into the circulation [10,75]. 

**Figure 3:**
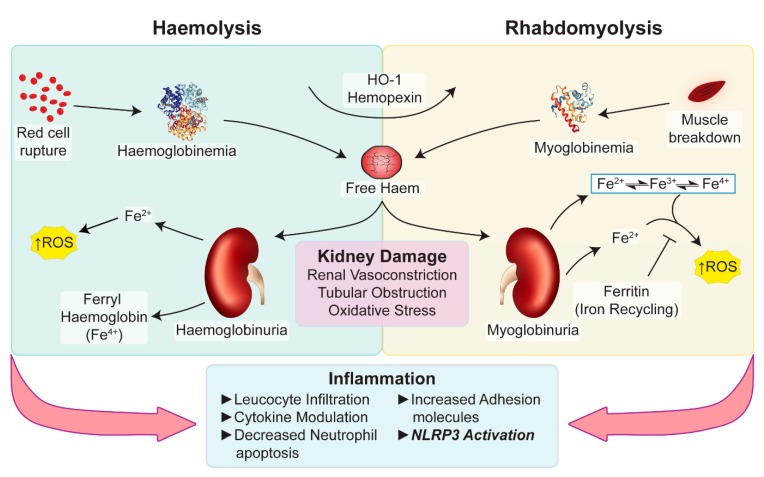
Potential pathways underlying haem-associated kidney injury. Free haem generated by rhabdomyolysis and haemolysis are effectively removed by HO-1 and hemopexin. The binding capacity of these proteins is saturated in pathological conditions and free haem continues to be present. Haemoglobin, myoglobin and plasma free-haem are freely filtered by the glomerulus and can be deposited within the tubules. Oxidative stress, renal vasoconstriction, tubular obstruction by casts, iron-mediated tubular toxicity and inflammation play an important role in acute pigment nephropathy. Myoglobin (PBD ID: 1MBN) and haemoglobin (PBD ID: 1BIJ) structures generated using the RCSB PDB NGL viewer [67]. NLRP3: nucleotide-binding domain-like receptor protein 3; HO-1: Haem Oxygenase-1; ROS: Reactive Oxygen Species.

Myoglobinuric AKI is the most severe complication of rhabdomyolysis [76]. Myoglobin is one of the pathogenic drivers of renal injury following rhabdomyolysis. Myoglobin is cytotoxic, activating both pro-oxidant and inflammatory pathways. Cytotoxicity is augmented in the presence of volume depletion and aciduria, common features of AKI [77,78]. Renal vasoconstriction, tubular obstruction and apoptosis are additional pathological processes in myoglobin toxicity, see Figure 3 [10,12,79]. 

There is a large volume of published studies describing oxidative stress in myoglobinuric AKI [10,12,13,80]. As for other hemoproteins, myoglobin possesses a haem centre that can catalyse the production of ROS within the kidneys. The haem group within myoglobin is capable of cycling between various oxidative states (ferrous = Fe^2+^; ferric = Fe^3+^; and ferryl = Fe^4+^) that may lead to lipid peroxidation independently of the Fenton reaction and iron release, see Figure 3 [12,13,80].

Most studies of the inflammatory pathogenic processes in myoglobinuric AKI are derived from experimental animal models and transformed cell lines. In a rat model of glycerol-induced rhabdomyolysis, macrophage infiltration was evident in the renal cortex as early as six hours following glycerol injection [79]. In vitro evidence suggests myoglobin polarizes macrophages toward both M1 (pro-inflammatory) and M2 (anti-inflammatory/pro-fibrotic) phenotypes, whilst in vivo research indicates that a reduction in oxidative stress may facilitate kidney tissue repair via a skewing of macrophages toward an M2 subtype [10,81].

Indeed, inflammation is involved in the pathogenesis of rhabdomyolysis-induced AKI, with emerging evidence of a functional role for the NLRP3 inflammasome in this disease process. Komada, et al. [17] reported greater expression of inflammasome-related molecules (NLRP3, ASC, caspase-1 and IL-1β) in the renal parenchyma following glycerol-induced myoglobinuric AKI [17]. Furthermore, activation of the inflammasome pathway correlated with leukocyte infiltration, tubular injury and dysfunction in the diseased kidney. Notably, these endpoints were markedly attenuated in *Nlrp3^-/-^, Asc^-/-^* and *Casp1^-/-^* knockout mice [17].

At present, many questions regarding the potential triggers of the inflammasome cascade in myoglobinuric AKI remain unanswered. Komada, et al. [17] carried out in vitro experiments using renal tubular epithelial cells incubated with hemin (the oxidised form of haem), ferrous and ferric myoglobin, all potential stimuli of the NLRP3 inflammasome in myoglobinuric AKI. Although these experimental data were not published, the authors reported that these stimuli were insufficient to activate NLRP3 [17]. Although innate immune cells (DC, macrophages) have the required components for canonical inflammasome activation [74,82], the ability of tubular epithelial cells to secrete mature IL-1β via this two-step process remains uncertain [58,74,82]. Therefore, the absence of inflammatory cells in the in vitro experiments of Komada et al. may explain why they failed to demonstrate triggering of canonical inflammasome activation.

Finally, as the pathogenesis of rhabdomyolysis is multifactorial, the role of other concomitant factors, acting either as priming stimuli or directly activating the NLRP3 inflammasome, should not be ignored. For instance, data from several studies suggest that different types of crystals, such as calcium oxalate, monosodium urate and cholesterol, can function as DAMPs to trigger NLRP3 inflammasome activation [22,25,83]. Recently, we highlighted a potential role for urate crystals in generating oxidative stress and activating the NLRP3 inflammasome in an animal model of rhabdomyolysis-associated AKI [10]. Thus, additional research is required to validate this hypothesis as well as to further elucidate the mechanisms underlying inflammation in human myoglobinuric AKI. 

## 5. Haemoglobin-Mediated Pigment Nephropathy

Haemolysis is defined as the rupture of red blood cells (RBC) as a result of intrinsic or extrinsic stresses, leading to the release of their intracellular contents, including hemoprotein haemoglobin [84]. Massive intravascular haemolysis is uncommon but occurs in life-threatening conditions such as poisoning, snake and insect envenomation, idiosyncratic drug reactions, haemolytic uraemic syndrome, paroxysmal nocturnal hemoglobinuria, malaria, haemorrhagic fevers, leptospirosis and septic shock [85,86,87,88,89,90,91].

In the event of haemolysis, plasma proteins such as haemoglobin-binding haptoglobin and haem-binding hemopexin effectively remove intravascular-produced haemoglobin/haem, thus mitigating haem-mediated deleterious effects [69]. However, under pathological conditions, the binding capacity of these plasma proteins is saturated, resulting in excess free haemoglobin in circulating blood [69]. Haemoglobulin and haem are filtered by the glomerulus, and free haemoglobin in the resultant ultrafiltrate is reabsorbed by the proximal tubules in an endocytic process involving the megalin-cubilin receptor system [65]. However, this absorption transport pathway is also concentration-dependent and large quantities of haemoglobin in the proximal tubules will saturate it, with free haemoglobulin/haem retained in the proximal tubules, leading to nephrotoxicity.

Many diseases featuring massive or recurrent haemolysis are complicated by AKI [9,64,84]. Prior to modern transfusion practices, ABO incompatibility was the most common cause of hemolysis-associated AKI [9]. With the exception of ABO-incompatible blood transfusions, haemolysis is now considered a contributing, rather than sole, trigger in the pathogenesis of haemoglobinuria-related AKI [9]. In fact, some conditions such as poisoning, envenomation and leptospirosis, may present with both haemolysis and rhabdomyolysis [85,92,93,94,95]. Furthermore, in malaria-associated AKI, other mechanisms play a greater pathogenic role than haemolysis, including mechanic obstruction by parasitized RBCs, the pro-inflammatory cytokine storm and immune-complex deposition [9,96].

The pathogenesis of haemoglobinuric AKI is multi-factorial, with aciduria, dehydration and renal ischaemia being the established contributing factors in the pathobiological processes [61,62]. These concomitant conditions are thought to enhance haem toxicity by favouring iron release and thus, pro-oxidant cytotoxic conditions [61]. 

As in myoglobin-derived PN, haemoglobin-derived free haem can drive oxidative stress, increased expression of adhesion molecules and elevated leukocyte infiltration into the diseased kidney [66,72]. Haemolysis also generates DAMP activity that triggers sterile inflammatory responses via the NLRP3 inflammasome [97]. In addition to haem, ruptured RBCs release heat shock proteins, ATP, IL-33 and mtDNA that are recognized triggers of the inflammasome cascade [97]. A correlate is found in humans with the disease of sickle cell, where patients commonly present with a state of chronic low-grade inflammation [69,98].

Intravascular hemolysis may also lead to haemoglobin in different oxidative states, i.e., hemoglobin (Fe^2+^), methemoglobin (Fe^3+^), and ferryl haemoglobin (Fe^4+^) [99]. Nyakundi, et al. [99] demonstrated both haem and ferryl haemoglobin stimulated LPS-primed macrophages to upregulate IL-1β mRNA and induce active IL-1β secretion. Further experiments conducted by Dutra et al. showed that the iron present within the haem molecule, not free iron, was the most important stimulus triggering the NLRP3 inflammasome and IL-1β secretion in macrophages and ultimately contributed to hemolysis-associated lethality [8]. Understanding these molecular pathways triggered by distinct haem motifs may prove useful in identifying novel therapeutic targets for haemoglobin/myoglobin-mediated pigment nephropathies.

## 6. Inflammasome Inhibition as a Potential Therapeutic Target

The significant pathological role of inflammasome activation in several chronic inflammatory diseases has made it an attractive target for therapeutic intervention. There are two approaches in current strategies inhibiting the inflammasome: (1) Targeting inflammasome activation directly and/or (2) targeting down-stream effects of IL-1β. Here, we review several compounds that could be repurposed, in combination with existing therapies, to ameliorate inflammatory immune responses in PN.

### 6.1. NLRP3 Inflammasome Inhibitors

Several compounds have been identified and developed for therapeutic inhibition of NLRP3 inflammasome activation. These established inflammasome-inhibiting compounds have been extensively reviewed by Lopez-Castejon and Pelegrin [100] and, more recently, by Baldwin, et al. [101]. Several preclinical studies have already investigated the use of these inflammasome inhibitors in AKI and CKD nephropathies, but their therapeutic efficacy has not been tested in PN.

The second-generation sulfonylurea drug, glyburide (also glibenclamide), is an established compound for the treatment of human type II diabetes mellitus [100,101]. Glyburide blocks K_ATP_ channels, depolarizing the cell membrane, triggering the release of insulin from pancreatic β-cells [100,101]. Glyburide’s actions were originally thought to be mediated via its role as a K_ATP_ channel blocker, but emerging evidence suggests that it, in fact, prevents the formation of ASC specks [101]. However, the specific mechanism of the interactions of glyburide and NLRP3 remain poorly understood. 

Glyburide has been used in an adenine-rich diet rat model of CKD. In this study, glyburide treatment attenuated NLRP3 expression, improved renal function and ameliorated the CKD histopathology [102]. Unfortunately, glyburide is generally not a recommended treatment in CKD patients due to the increased risk of hypoglycemia [103]. In addition, patients with glucose-6-phosphate dehydrogenase deficiency are susceptible to developing haemolytic anemia following glyburide treatment [104,105].

A novel subclass of sulfonylurea containing compounds, derived from glyburide, was identified by Perregaux, et al. [106]. These compounds inhibited post-translational processing of IL-1β, resulting in little-to-no maturation or extracellular release of the cytokine. One of these compounds, MCC950 (also CP-456,773), was reported by Coll, et al. [107] as a potent, specific inhibitor of the NLRP3 inflammasome. Whilst the mechanism of MCC950-mediated NLRP3 inhibition is still poorly understood, MCC950 has been studied in several disease models, including colitis [108], Parkinson’s disease [109], diabetic encephalopathy [110] and non-alcoholic steatohepatitis [111]. Recent studies also evaluated MCC950 in pre-clinical models of AKI and CKD. MCC950 treatment attenuated kidney fibrosis in a murine model of diet-induced oxalate crystal-nephropathy [20]. Furthermore, MCC950 treatment abrogated kidney damage and ameliorated systemic blood pressure in a murine model of hypertension, induced by both surgery (uninephrectomy) and treatment with deoxycorticosterone [60]. MCC950’s relatively short half-life and its specificity for the NLRP3 inflammasome [107] make it, and its derivatives, ideal candidates for further investigations in PN. 

### 6.2. Anti-IL-1β and IL-1 Receptor Antagonists

Inhibition of the down-stream IL-1β-signalling pathways has been widely adopted in rheumatology for treatment of auto-inflammatory diseases [112]. Strategies for these therapies involve: (1) Reducing the amount of IL-1β available for activating the endogenous IL-1 receptor (IL-1R) or (2) inhibiting the endogenous receptor directly. 

Canakinumab is a potent monoclonal antibody specific for IL-1β [113,114] and an established therapeutic in the treatment of rheumatoid arthritis [112]. Canakinumab has been evaluated in patients with CKD, reducing the risk of major adverse cardiovascular event rates among high-risk atherosclerosis patients [115]. However, no differences in kidney function (as measured by the estimated Glomerulus Filtration Rate) were reported between placebo and Canakinumab-treated CKD patients [115]. A common CKD co-morbidity is gout, which arises as a consequence of increased uric acid. Inflammasome activation is imputed to be the prime mechanism of this auto-inflammatory condition [25]. A clinical trial using Canakinumab showed significantly reduced rates of gout attacks in patients, although no changes in serum uric acid concentrations were observed [116]. Studies such as these provide important foundational evidence for further pre-clinical studies of Canakinumab for the treatment of PN. 

Therapeutic strategies targeting the IL-1 receptor (IL-1R) are also used in current clinical practice. Anakinra is a recombinant human IL-1R antagonist, competing with IL-1β for binding with the IL-1R [117]. Anakinra is another established therapeutic in the treatment of auto-inflammatory diseases in rheumatology. Notably, it has been successfully used in patients with Familial Mediterranean Fever (FMF) [118,119], an auto-inflammatory disease associated with mutations in the inflammasome component pyrin that results in triggering inflammasome activation [120,121]. The therapeutic use of anakinra for treating acute gout attacks in CKD patients is currently in clinical trials (ASGARD study), with the results yet to be published [117]. Interestingly, anakinra is being investigated as a third-line therapy in this ASGARD study, following non-response to second-line therapy, where the development of rhabdomyolysis was a reported side-effect [117,122]. 

Although these IL-1β- and IL-1R-targeting drugs are proving to be effective inflammasome inhibitors, pre-clinical studies investigating their efficacy for the treatment of PN are yet to be performed. These studies need to include in vivo and in vitro models of PN to not only establish therapeutic efficacy but also any unforeseen off-target effects. 

## 7. Concluding Remarks

The release of haem by myoglobin and haemoglobin catabolism is pivotal in the pathogenesis of PN. Whilst haem toxicity is clinically recognized as important, the role of haem in the mechanism of the associated kidney inflammation may be overlooked. Irrespective of its source, haem triggers NLRP3 inflammasome activation, but this mechanistic pathway of disease in PN is still poorly understood. Contemporary studies have shifted to the role of haem driving kidney inflammation via NLRP3 inflammasome activation. The research is focused on the canonical activation of the inflammasome within immune cell populations by haem. The non-canonical activation of the inflammasome in immune cell populations by haem has not been investigated. Furthermore, neither canonical nor non-canonical mechanisms of inflammasome activation within kidney parenchymal cells are fully understood. Well-designed studies are required to address both, focusing on haemolytic driven AKI for which there is currently a lack of information. 

The aim of future PN research is to provide evidence to move to pre-clinical studies of potential treatments for both myoglobinuric and haemolytic AKI. Non-renal studies with IL-1R antagonists and direct NLRP3 inflammasome inhibitors are advanced, with small molecules in clinical use for auto-immune rheumatological diseases. Several pre-clinical studies have investigated their therapeutic role in different patterns of kidney disease, but not PN. These studies provide the rationale for translation into clinical trials for the prevention and treatment of PN.

## Abbreviations

AKI	Acute Kidney Injury
ASC	Apoptosis-associated speck-like protein containing a CARD domain
ATP	Adenosine Triphosphate
CARD	Caspase activation and recruitment domain
CD	Cluster of differentiation
CKD	Chronic Kidney Disease
DAMPs	Damage-associated molecular patterns
DC	Dendritic cells
DKD	Diabetic kidney disease
ESCRT	Endosomal sorting complexes required for transport
FMF	Familial Mediterranean Fever
GSDMD	Gasdermin-D
HAMPs	Homeostasis-altering molecular processes
HO	Haem Oxygenase
HO-1	Haem Oxygenase-1
ICAM-l	Intercellular Adhesion Molecule-1
IL	Interleukin
IL-1R	IL-1 receptor
LPS	Lipopolysaccharide
MAVS	Mitochondrial antiviral signal
mtDNA	Mitochondrial DNA
NADPH	Dihydronicotinamide-adenine dinucleotide phosphate
NLRP3	Nucleotide-binding domain-like receptor protein 3
PAMPs	Pathogen-associated molecular patterns
PN	Pigment Nephropathy
PRRs	Pattern recognition receptors
PTEC	Proximal Tubule Epithelial Cells
RBC	Red Blood Cells
ROS	Reactive Oxygen Species
TLRs	Toll-like receptors
UUO	Unilateral ureteral obstruction
VCAM-l	Vascular Adhesion Molecule-1
XO	Xanthine Oxidase
